# Shotgun metagenomic profiling reveals *Bacillus*-dominated bacterial communities in urban rooftop and surface garden soils of Bangladesh

**DOI:** 10.1371/journal.pone.0344114

**Published:** 2026-03-06

**Authors:** M. Nazmul Hoque, Md. Liton Rana, Md Abu Ahsan Gilman, Pritom Kumar Pramanik, Md. Saiful Islam, Sadia Afrin Punom, Rabith Rahman, Jayedul Hassan, Tofazzal Islam, Srinivasan Ramasamy, Pepijn Schreinemachers, Ricardo Oliva, Md. Tanvir Rahman

**Affiliations:** 1 Molecular Biology and Bioinformatics Laboratory, Department of Gynecology, Obstetrics and Reproductive Health, Gazipur Agricultural University, Gazipur, Bangladesh; 2 Department of Microbiology and Hygiene, Faculty of Veterinary Science, Bangladesh Agricultural University, Mymensingh, Bangladesh; 3 National Engineering Research Center of Industrial Wastewater Detoxication and Resource Recovery, Research Center for Eco-Environmental Sciences, Chinese Academy of Sciences, Beijing, China; 4 University of Chinese Academy of Sciences, Beijing, China; 5 Department of Animal Sciences, University of California – Davis, Davis, California, United States of America; 6 Department of Computer Science, Toronto Metropolitan University, Toronto, Canada; 7 Institute Biotechnology and Genetic Engineering, Gazipur Agricultural University, Gazipur, Bangladesh; 8 World Vegetable Center, Shanhua, Tainan, Taiwan; 9 World Vegetable Center, Bangkok, Thailand; DIU: Dhaka International University, BANGLADESH

## Abstract

Urban rooftop and surface garden systems play a critical role in food security in densely populated regions, yet their soil microbiomes remain understudied. To date, no baseline data exists on rooftop and surface garden soil microbiomes in Bangladesh. Understanding these communities is vital for enhancing soil health, nutrient cycling, and resilience for sustainable, climate-adapted urban agriculture. This study therefore investigated the bacterial diversity and community structure of rooftop and surface garden soils across Dhaka and Gazipur, Bangladesh. The goal was to uncover location- and garden-type-specific patterns that influence soil functionality. Using shotgun metagenomics of 27 garden soil samples (seven Dhaka rooftop [DRG], six Dhaka surface [DSG], eight Gazipur rooftop [GRG], and six Gazipur surface [GSG]), we identified 755 bacterial species dominated by Firmicutes (65–83%) and Proteobacteria (3–25%). While alpha diversity was consistent across sites (p > 0.05), beta diversity revealed distinct community structuring (p = 0.017), with surface gardens harboring greater bacterial richness (DSG:717, GSG:750 species) and elevated Bacteroidota (DSG:11.5%, GSG:2.7%) compared to rooftop soils. Strikingly, *Bacillus* species dominated all soils (>53% relative abundance) but exhibited location-specific distributions. DRG soils were notably enriched with *B. paralicheniformis* (28.3%) and *B. licheniformis* (25.2%). In contrast, DSG was characterized by *B. cereus sensu lato* (16.0%), *Brevibacillus agri* (12.1%), and *Flavobacterium thermophilum* (11.4%). GRG soils were dominated by *B. cereus sensu lato* (42.4%) and *B. agri* (11.5%). GSG soils showed diverse *Bacillus* species, including *B. stratosphericus* (14.6%), *B. licheniformis* (12.7%), *B. safensis* (9.7%), and *B. altitudinis* (8.8%). Of 41 detected *Bacillus* species, more than 58.0% were shared across gardens, yet their abundances varied with microhabitat. Moreover, KEGG profiling revealed marked functional divergence among urban garden soils. Carbohydrate metabolism dominated all sites (9.30–11.07%). DRG was uniquely enriched in photosynthesis (8.40%) and methane metabolism (8.62%), whereas DSG, GRG, and GSG showed higher oxidative phosphorylation (3.75–4.08%), two-component systems (3.24–3.73%), and biosynthetic pathways. This study unveils the ecological dominance of *Bacillus* species in urban agricultural soils, with location-driven compositional and functional shift. These findings are pivotal for optimizing sustainable urban agriculture in rapidly developing regions, where soil bacteriomes can be harnessed to improve crop resilience and food security.

## 1. Introduction

Urbanization profoundly alters land use, impacting surface landscapes as well as the physicochemical and microbial properties of soil [1–3]. These changes threaten soil biodiversity and the biological processes essential for ecosystem functionality [4]. Soil microorganisms are integral to these functions, driving nutrient cycling, decomposing organic matter, maintaining plant diversity, enhancing soil structure, and suppressing diseases [5,6]. This critical link between soil health and the well-being of plants, animals, and humans is increasingly compromised by anthropogenic activities [7–9]. The soil microbiome, crucial for nutrient cycling, organic matter decomposition, and plant health [10], enhances fertility and crop productivity through processes like nitrogen fixation and phosphate solubilization [11]. Beneficial microbes further contribute by suppressing pathogens, improving drought resilience, and aiding phytoremediation [12]. A diverse microbiome thus underpins sustainable agriculture, carbon sequestration, and biodiversity conservation [13]. Within this microbiome, the genus *Bacillus* is one of the most ubiquitous and ecologically significant bacterial genera [14]. Its species exhibit strong genetic and metabolic plasticity, enabling them to persist in heterogeneous environments and contribute to key functions such as nutrient turnover, nitrogen availability, and plant stress tolerance [15–17]. A defining trait of *Bacillus* spp. is their ability form resilient endospores, which confer resistance to extreme stresses including heat, UV radiation, and desiccation, and facilitate long-term survival and global dispersal [15,16,18]. Ecologically, *Bacillus* spp. drive nutrient cycling, organic matter decomposition, and pathogen suppression through antimicrobial production [18]. Their resilience allows them to dominate microbial communities even in extreme environments like acidic, saline-alkaline, and desert soils [19–21]. These capabilities underscore their broad biotechnological relevance, with applications in agriculture as biofertilizers and biopesticides, in industrial enzyme and biofuel production, in environmental remediation, and as probiotics [14,20,22]. Given their ecological significance and applied potential, considerable research has focused on isolating and characterizing *Bacillus* strains from diverse environments and mapping their distribution [19,20,23,24]. As resilient and functionally versatile bacteria, *Bacillus* spp. represent a critical link between microbial ecology and innovative, sustainable biotechnological applications. Despite their critical role in delivering ecosystem services, urban soils have not received much attention in agricultural research.

Urban agriculture, including rooftop and surface gardening, has emerged as a sustainable strategy to address food security, environmental challenges, and the adverse effects of urbanization, such as the urban heat island effect. Rooftop gardens, in particular, provide a wide array of benefits that extend beyond mere food production [25,26]. They serve as productive spaces for cultivating fresh vegetables, herbs, and spices, offering urban residents access to nutritious, locally grown produce. Beyond their agricultural value, rooftop gardens create vibrant social and recreational spaces, fostering community interaction and providing a respite from the dense urban environment [25,26]. These green spaces can also act as therapeutic environments, promoting mental well-being and offering opportunities for relaxation and connection with nature. Additionally, rooftop gardens play a crucial role in mitigating urban environmental challenges. They help reduce the urban heat island effect by cooling buildings and their surroundings, while also improving air quality by filtering pollutants and capturing carbon dioxide. By integrating greenery into urban landscapes, rooftop gardens contribute to more sustainable and liveable cities, addressing both environmental and social needs [13,14]. In Bangladesh, rooftop gardening is becoming increasingly popular, yet the microbial ecology of these systems remains poorly understood. The potential for microbial contamination of fruits and vegetables grown in these gardens poses a significant health risk, particularly in densely populated urban areas where food safety is a growing concern [27].

Metagenomics is a foundational tool for comprehensively studying microbial communities and their functional dynamics within complex environments. Through the direct sequencing of genomic DNA from samples like soil, water, sediments, milk, milk products, fish, and host-associated niches, this approach facilitates the identification of uncultivated microorganisms and clarifies their ecological roles [28–32]. *16S rRNA* gene amplicon sequencing provides a cost-effective overview of bacterial and archaeal community composition, making it suitable for large-scale surveys of taxonomic diversity [3,33,34]. However, it offers limited resolution at the species level and provides little direct information on microbial functions. In contrast, shotgun metagenomics sequencing involves all genetic material present in soil, enabling comprehensive profiling across bacteria, archaea, fungi, and viruses [32,35,36], while simultaneously revealing functional potential through metabolic pathway reconstruction related to nutrient cycling, organic matter decomposition, and plant growth promotion [9,37]. Although shotgun metagenomics is more computationally intensive and costly, it delivers deeper ecological and functional insight. Beyond taxonomy, functional profiling of microbial communities provides critical insights into host–microbe–environment interactions by revealing the metabolic potential and ecological roles of resident microorganisms [35,38]. Shotgun metagenome sequencing enables comprehensive characterization of community-wide functional genes, allowing the identification of pathways involved in nutrient cycling, energy metabolism, stress response, and host adaptation [9,39]. Such functional resolution is particularly valuable for understanding how microbial communities respond to environmental gradients and anthropogenic pressures, and how these shifts may influence host health and ecosystem stability. Bioinformatics tools such as KEGG (Kyoto Encyclopedia of Genes and Genomes) pathway annotation allow inference of metabolic and ecological functions encoded within the microbiome, including energy metabolism, transport systems, and host-associated processes [29,36,40].

Urban agriculture is rapidly expanding in response to population growth and food security challenges, yet a significant knowledge gap persists regarding the microbial ecology of rooftop and surface garden soils in regions like Bangladesh. To date, no baseline metagenomic data exist for these systems. While earlier studies have characterized urban garden soil bacterial communities using *16S rRNA* gene amplicon sequencing [3,41], comprehensive shotgun metagenomic investigations of urban gardening ecosystems in Bangladesh are lacking. Therefore, this study investigates the bacteriome composition and diversity, and KEGG functional pathway variations in urban garden soils from Dhaka and Gazipur, Bangladesh, encompassing both densely populated and peri-urban environments. Shotgun whole metagenome sequencing (WMS) was used to characterize bacterial communities and their functional potential. Such insights are essential for improving soil health, supporting crop productivity, and promoting sustainable urban agriculture in Bangladesh and similar environments.

## 2. Materials and methods

### 2.1 Ethical approval and informed consent

This study did not involve human participants; therefore, ethical approval and informed consent were not required.

### 2.2 Study areas and sample collection

**Fig 1:**
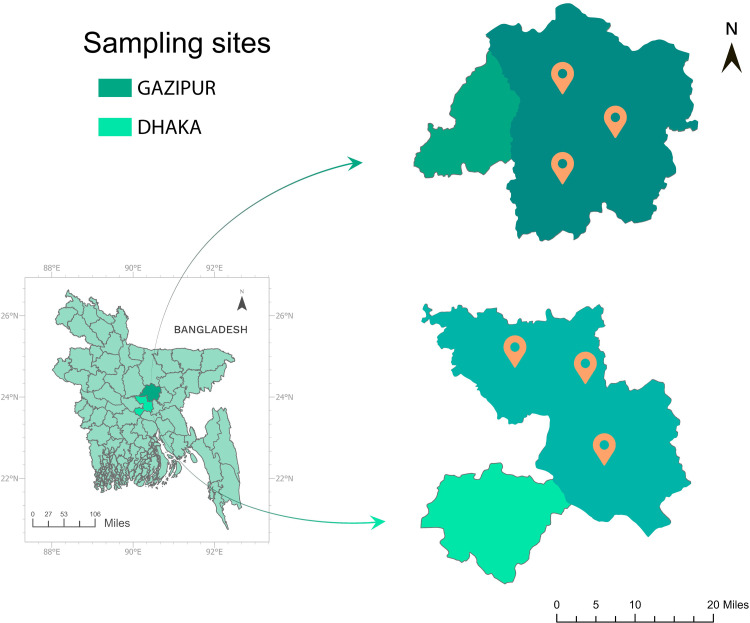
Study area map represents the sampling sites. **(A)** Gazipur district **(B)** Dhaka district- created on ArcGIS Pro 10.7 (ESRI, USA) based on Geographical Information System (GIS).

This study was conducted using 27 soil samples collected from 27 gardens in the Dhaka and Gazipur districts of Bangladesh, comprising seven rooftop gardens in Dhaka (DRG), six surface gardens in Dhaka (DSG), eight rooftop gardens in Gazipur (GRG), and six surface gardens in Gazipur (GSG). The selection criteria were based first on garden type (*e.g.,* rooftop vs. surface) and then on location (*e.g.,* urban vs. peri-urban). The study areas (**Fig 1**), located approximately 15–20 km apart (coordinates: 23.72–23.99° N and 90.40–90.42° E), experience a tropical wet and dry climate with an average annual rainfall of 1875 mm, a mean annual temperature of 26.1 °C, and humidity of 65.8% [25]. Most rooftop gardens utilized a specially prepared soil mixture of compost and woodchips, enriched with organic manure as a biofertilizer to enhance nutrient availability, soil structure, and water retention for growing leafy vegetables and green fruits. The study involved non-invasive soil sampling from private gardens with no involvement of human subjects or personal data collection beyond basic garden characteristics. According to the national ethical guidelines of Bangladesh and the policies of Bangladesh Agricultural University, no specific institutional review board (IRB) permit was required for garden soil (environmental) sampling. However, prior to sample collection, the purpose of the study and procedures were clearly explained to all participating farm owners, from whom verbal informed consent was obtained for the collection of soil samples from their gardens. Composite soil samples were collected during the morning hours in April and July 2024 from multiple representative locations within each garden. Using a hand trowel, subsamples were collected aseptically from a depth of 0–5 cm with a 2.5 cm diameter tool [3], mixed thoroughly in clean containers, and labelled for analysis. Each composite sample was air-dried, homogenized, sieved through a 6.35 mm mesh to remove debris, following standard microbiological soil sampling protocols [13,42]. The samples were subsequently divided into two 1.0 g technical replicates [3], with one set stored at −80 °C as a backup. This standardized protocol ensured consistency and reliability for subsequent bacterial community analysis.

### 2.3 DNA extraction and shotgun whole metagenome sequencing

DNA of the collected soil samples was extracted using the Qiagen DNeasy PowerSoil Kit (QIAGEN, Hilden, Germany) following the manufacturer's instructions. DNA quantity and purity were determined using a NanoDrop ND-2000 spectrophotometer (ThermoFisher, USA) by measuring the 260/280 absorbance ratio [9]. Shotgun whole metagenome sequencing (WMS) libraries were prepared with Nextera XT DNA Library Preparation Kit [9,29], according to the manufacturer’s instructions. The library quality was assessed on the Qubit 2.0 Fluorometer (Thermo Scientific) and Agilent Bioanalyzer 2100 system. Paired-end (2  ×  150 bp) sequencing was performed on a NovaSeq 6000 sequencer (Illumina Inc., USA) from the Macrogen Inc. (www.macrogen.com) Seoul, Republic of South Korea.

### 2.4 Bioinformatics analysis

The generated FASTQ files were concatenated, and processed using Trimmomatic v0.39 (with parameters: k = 21, mink = 6, ktrim = r, ftm = 5, qtrim = rl, trimq = 20, minlen = 30, overwrite = true) [43] to eliminate Illumina adapters, known Illumina artifacts, and phiX sequences. Reads failing to meet the specified quality thresholds or containing more than one ‘N’ were excluded from further analysis. The DNA from 27 samples generated 1,345.11 million raw reads with an average of 49.82 million (maximum = 66.34 million, minimum = 42.07 million) reads per sample (S1 Table). High-quality reads were analyzed using a combination of open-source, cloud-based metagenomic mapping and assembly-based hybrid approaches through CZ ID [44]. CZ ID, an open-source cloud-based pipeline, was employed for taxonomic classification of bacterial sequences, applying thresholds of NTL (alignment length in base pairs) ≥ 50 and NT percent identity ≥ 90. For CZ ID analysis, a custom ‘target’ genome library was created, incorporating all bacterial sequences from the NCBI database. The WMS reads were aligned to the target library using the highly sensitive Bowtie 2 algorithm [45]. Reads aligning to the human genome (hg38) and non-bacterial organisms (*e.g.,* archaea, viruses, and fungi) were filtered out. Alpha diversity, which measures diversity within samples, was assessed using the Shannon, Simpson, Observed species, and Chao1 diversity indices [46] based on CZ ID read assignments and counts. To visualize variations in bacteriome diversity, principal coordinate analysis (PCoA) was conducted using the Bray-Curtis distance method [47], and non-metric multidimensional scaling (NMDS) was performed using weighted-UniFrac distance on CZ ID data at the bacterial species level. These analyses were performed using the Phyloseq R package, version 4.4 [48]. Data processing and visualization were executed through the Phyloseq R package [35] and ggplot2 [49], respectively. Furthermore, quality-filtered reads from each sample were *de novo* assembled individually using MEGAHIT version 1.2.9 [50]. Open reading frames were predicted from the assembled contigs using Prodigal version 2.6.3 [51]. Predicted protein sequences were aligned against the KEGG database (release 2021-05-01) [40] using DIAMOND version 2.0.11, with a stringent e-value threshold of 1e-5 [52]. KEGG Orthology (KO) identifiers were assigned, and reads were mapped back to the contigs to quantify the abundance of each KO in each sample. The relative abundance of KEGG pathways (at Level 3) was calculated by aggregating the abundances of associated KOs for each sample.

### 2.5 Statistical analysis

The Shapiro-Wilk test was employed to assess the normality of the data. For evaluating differences in the relative percent abundance of taxa between the garden types (rooftop and surface) and locations (Dhaka and Gazipur), the non-parametric Kruskal-Wallis test was utilized [36]. This statistical method was chosen due to its suitability for non-normally distributed data. Statistical analyses for the WMS data were initially conducted using embedded statistical tests within the pipeline (*i.e.,* Phyloseq) and subsequently validated using SPSS (Version 25.0, IBM Corp., NY, USA) with the aforementioned tests.

## 3. Results

### 3.1 Bacteriome diversity in rooftop garden and surface garden soils

**Fig 2:**
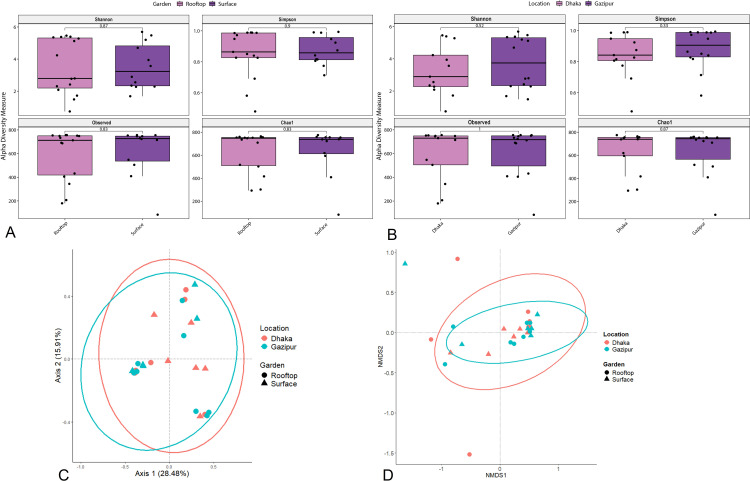
Shotgun metagenomics reveals the lower diversity of soil microbial communities in rooftop and surface gardens across Dhaka and Gazipur, Bangladesh. **(A-B)** Alpha diversity (within-sample) is analyzed based on (A) garden type and (B) location, with Shannon, Simpson, Observed, and Chao1 indices illustrating microbial richness and evenness in rooftop and surface garden soils. Boxplots display the results, and pairwise Kruskal-Wallis tests assess statistical differences, with p-values indicating no significance differences (*p* > 0.05). **(C-D)** Beta diversity (between-sample) reflects microbial community differences by garden type and location, measured using Bray-Curtis dissimilarity. The data is visualized through (C) principal coordinate analysis (PCoA) and (D) non-metric multidimensional scaling (NMDS), where samples are color-coded by garden type and location, with ellipses representing group clustering. The percentage of variation along the X and Y axes highlights the extent to which garden type and location shape microbial diversity.

To explore the diversity and composition of the bacteriomes, we conducted shotgun WMS on 27 soil samples collected from rooftop and surface gardens in Dhaka and Gazipur districts of Bangladesh. Detailed information on the sampling, demographics, WMS data, taxonomic units assigned per sample, and sequence read archive (SRA) accession numbers are provided in S1 Table. The WMS analysis of the 27 soil samples yielded a total of 1,345,113,860 raw reads, averaging 49,819,032 reads per sample. After quality filtering, 27,809,614 (out of 1,325,107,908 reads) high-quality reads (2.1% of the total) were mapped to 766 taxa representing microbial taxa, with an average GC content of 51.91% of the mapped reads (S1 Table). At the domain level, bacteria were the most abundant community, with an average abundance of 99.68%, followed by archaea (0.18%), and viruses (0.14%). This comprehensive dataset provides a robust foundation for understanding the bacterial community structure and diversity across the sampled environments. To assess variation in garden soil bacterial diversity across the studied metagenomes (*i.e.,* DRG, DSG, GRG, and GSG), we analyzed both within-sample (alpha) and across-sample (beta) diversity of the detected bacterial communities (**Fig 2**). Alpha diversity was assessed using Shannon, Simpson, Observed taxa, and Chao1 indices. No significant differences were observed between sample types (rooftop vs. surface garden soils) (p > 0.05, Kruskal-Wallis test) (**Fig 2A**) or between locations (Dhaka vs. Gazipur) (p > 0.05, Kruskal-Wallis test) (**Fig 2B**). These results indicate similar bacterial diversity across both sample categories and locations. Beta diversity, analyzed through PCoA using Bray-Curtis dissimilarity, revealed significant differences in bacterial community composition among the groups (p = 0.017, Kruskal-Wallis test). The PCoA plot showed distinct clustering of samples by location (Dhaka, Gazipur) and garden type (Rooftop, Surface), with Axis1 accounting for 28.48% and Axis2 for 15.91% of the variance (**Fig 2C**). The NMDS plot also revealed significant (p = 0.023, Kruskal-Wallis test) clustering of bacterial communities by location (Dhaka, Gazipur) and garden type (rooftop, surface), consistent with PCoA results (**Fig 2D**). In this study, we identified 19, 31, 75, 122, 275, and 755 bacterial phyla, classes, orders, families, genera, and species, respectively in the soils of both rooftop and surface gardens in Dhaka and Gazipur. Although the bacterial composition at higher taxonomic levels (phyla to families) showed no significant variation across DRG, DSG, GRG, and GSG metagenomes (S1 and S2 Figs), significant differences (p < 0.05, Kruskal-Wallis test) were observed in bacteriome diversity, composition, and relative abundances at the genus and species levels.

### 3.2 Bacterial community dynamics in rooftop and surface garden soils

**Fig 3:**
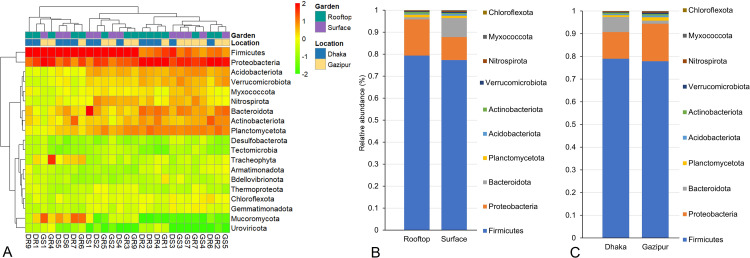
Taxonomic composition of soil microbiomes at the phylum level. **(A)** Heatmap illustrating the average relative abundance and hierarchical clustering of microbial phyla in soil samples from rooftop and surface gardens in Dhaka and Gazipur district of Bangladesh. The color bar represents the presence and completeness of each microbial phylum, ranging from −2 (lowest abundance) to 2 (highest abundance). Red cells indicate higher abundance, while green cells signify lower abundance within each sample. The colored squares at the top reflect the relative abundance of microbial phyla in individual samples. Soil sample labels: Dhaka rooftop (DR), Dhaka surface (DS), Gazipur rooftop (GR), and Gazipur surface (GS). **(B-C)** Stacked bar plots depicting the relative abundance and distribution of microbial phyla, arranged from bottom to top in descending order of abundance. Each plot shows the phylum-level microbial composition for specific sample types and locations.

**Fig 4:**
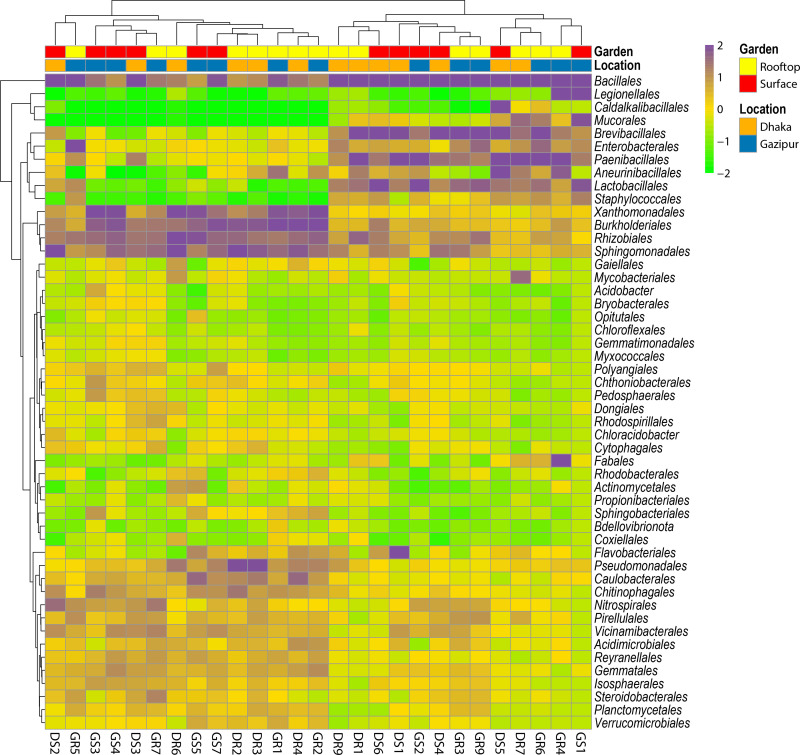
The taxonomic composition of soil microbiomes at the order level is presented through a heatmap, depicting the average relative abundance and hierarchical clustering of the top 50 bacterial orders in soil samples from rooftop and surface gardens in Dhaka and Gazipur, Bangladesh. The color bar of the heatmap indicates the relative abundance and completeness of each bacterial order, ranging from −2 (lowest) to 2 (highest). Purple cells signify higher bacterial abundance, while green cells indicate lower abundance across samples. Colored squares at the top of the heatmap reflect the relative abundance of bacterial orders in individual samples. Soil sample labels are denoted as Dhaka rooftop (DR), Dhaka surface (DS), Gazipur rooftop (GR), and Gazipur surface (GS).

We identified 19 bacterial phyla in soil samples collected from both rooftop and surface gardens in both locations (Dhaka and Gazipur). **Fig 3** illustrates the taxonomic composition of soil bacteriomes in rooftop and surface gardens from Dhaka and Gazipur, highlighting the relative abundance of dominant bacterial phyla. Firmicutes (65.30–83.16%) and Proteobacteria (3.42–24.92%) were the most prevalent phyla across all metagenomes, followed by Actinobacteria, Bacteroidota, and Acidobacteriota (**Fig 3A**), demonstrating their adaptability to urban and peri-urban soil environments. Notably, Bacteroidota exhibited significantly (p = 0.015, Kruskal-Wallis test) higher relative abundances in surface garden samples from Dhaka (DSG; 11.54%) and Gazipur (GSG; 2.69%) compared to rooftop gardens (DRG; 1.2% and GRG; 0.73%). Less abundant phyla, such as Chloroflexota and Verrucomicrobiota, were also detected and may play niche-specific ecological roles (S3 Fig.). Variations in bacterial diversity between gardening systems (rooftop vs. surface) (**Fig 3B**) and locations (Dhaka vs. Gazipur) (**Fig 3C**) reflect differences in environmental conditions, including soil quality and anthropogenic influences. Analysis of bacterial orders revealed distinct microbial community structures influenced by location and gardening systems. Significant variations (p = 0.012, Kruskal-Wallis test) were observed in taxonomic relative abundances. Across 27 metagenomic samples, a total of 75 bacterial orders were identified. *Bacillales* dominated across all environments, with relative abundances of 59.70% in DRG, 58.77% in DSG, 62.28% in GRG, and 60.85% in GSG, indicating its adaptability to diverse conditions (**Fig 4**, S1 Data). *Brevibacillales* showed notable enrichment in DSG (17.62%) and GRG (17.39%), but its presence was minimal in GSG (0.14%). Similarly, *Paenibacillales* was more prevalent in DRG (9.87%) compared to other locations (1.95–3.25%). *Flavobacteriales* was significantly abundant in DSG (11.27%) but nearly absent in other sites (<1%), whereas *Xanthomonadales* was enriched in GSG (7.60%) but scarce in DSG (0.16%). The remaining bacterial orders had lower relative abundances (< 1.0%) across these metagenomes (**Fig 4**, S1 Data), indicating a less prominent role in shaping the overall bacterial community structure.

**Fig 5:**
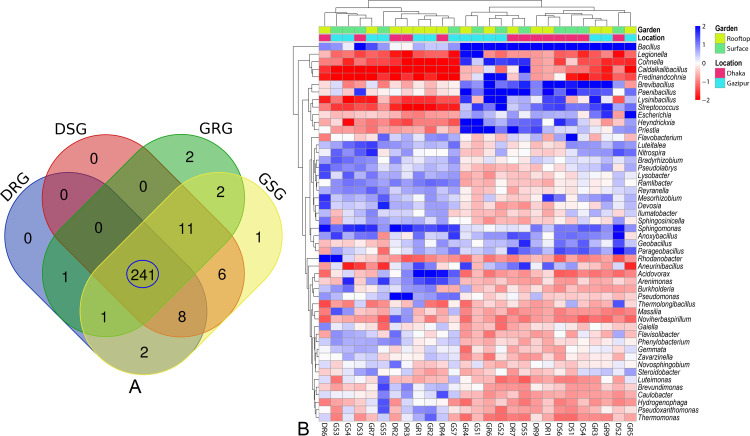
Taxonomic profile of microbiomes at genus level. **(A)** Venn diagrams showing the unique and shared composition of bacterial genera in Dhaka rooftop garden (DRG), Dhaka surface garden (DSG), Gazipur rooftop garden (GRG) and Gazipur surface garden (GSG) soil samples. Shared microbial taxa are highlighted in blue circle. **(B)** The taxonomic profile of soil microbiomes at the genus level is illustrated through a heatmap, representing the average relative abundance and hierarchical clustering of the 50 most prevalent bacterial genera in soil samples from rooftop and surface gardens in Dhaka and Gazipur, Bangladesh. The color gradient in the heatmap indicates genus abundance and distribution, ranging from −2 (lowest) to 2 (highest). Blue cells correspond to genera with higher relative abundance, while red cells denote lower abundance. Colored squares positioned above the heatmap reflect the relative abundance of bacterial genera within individual samples. Soil sample designations include Dhaka rooftop (DR), Dhaka surface (DS), Gazipur rooftop (GR), and Gazipur surface (GS).

**Fig 6:**
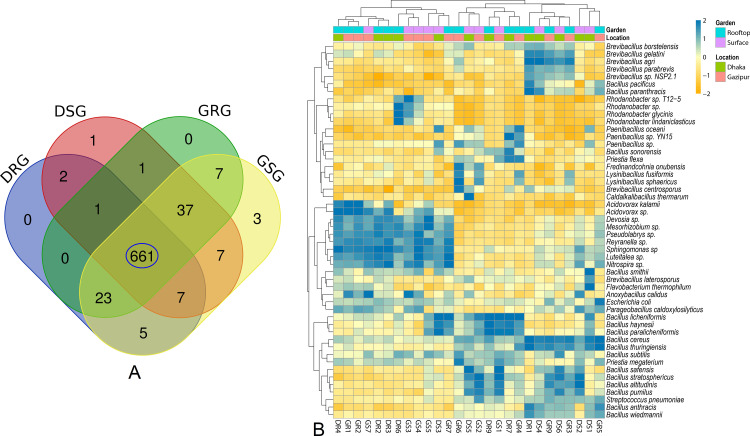
Taxonomic profile of microbiomes at species level. **(A)** Venn diagrams showing the unique and shared composition of bacterial species in Dhaka rooftop garden (DRG), Dhaka surface garden (DSG), Gazipur rooftop garden (GRG) and Gazipur surface garden (GSG) soil samples. Shared microbial species are highlighted in blue circle. **(B)** The taxonomic composition of microbiomes at the species level is visualized through a heatmap, illustrating the average relative abundance and hierarchical clustering of the 50 most dominant bacterial species in soil samples from rooftop and surface gardens in Dhaka and Gazipur, Bangladesh. The color scale of the heatmap represents species abundance and completeness, ranging from −2 (lowest) to 2 (highest). Deep sea blue cells indicate species with higher relative abundance, while orange cells signify lower abundance across samples. Colored squares above the heatmap correspond to the relative abundance of bacterial species in individual samples. Soil sample labels are designated as Dhaka rooftop (DR), Dhaka surface (DS), Gazipur rooftop (GR), and Gazipur surface (GS).

Our analysis revealed significant differences (p = 0.023, Kruskal-Wallis test) in both the composition and relative abundances of bacterial taxa at the genus level according to sample categories (rooftop vs. surface) and locations (Dhaka vs. Gazipur). Out of 275 bacterial genera, 253, 266, 258, and 272 were detected in DRG, DSG, GRG, and GSG soil samples, respectively (**Fig 5A**). Notably, 84.0% (231/275) of the genera were shared across all metagenomes (DRG, DSG, GRG, and GSG). *Bacillus* was the most dominant genus in the soil sample across all metagenomes, with relative abundances of 58.21% in DRG, 54.47% in DSG, 54.94% in GRG, and 55.39% in GSG soils. In DRG soil samples, other predominant bacterial genera included *Paenibacillus* (9.84%), *Brevibacillus* (4.53%), *Rhodanobacter* (3.88%), *Sphingomonas* (3.15%), *Pseudomonas* (2.25%), and *Acidovorax* (1.50%). Conversely, DSG soils also exhibited higher proportions of *Brevibacillus* (18.02%), *Flavobacterium* (11.52%), *Paenibacillus* (3.06%), *Parageobacillus* (1.52%), *Sphingomonas* (1.39%), and *Anoxybacillus* (1.38%) (**Fig 5B**). The GRG soils showed significant abundances of *Brevibacillus* (17.55%), *Lysinibacillus* (3.61%), *Acidovorax* (2.92%), *Paenibacillus* (1.65%), *Escherichia* (1.37%), *Priestia* (1.17%), and *Sphingomonas* (1.10%). In contrast, the GSG soils were characterized by higher proportions of *Rhodanobacter* (3.02%), *Paenibacillus* (2.17%), *Lysinibacillus* (2.13%), *Arenimonas* (1.97%), *Devosia* (1.67%), *Streptococcus* (1.59%), *Sphingomonas* (1.55%), and *Anoxybacillus* (1.02%). Other genera were present in lower abundances (< 1.0%) across all soils (**Fig 5B**, S1 Data). We further explored whether the species-level composition and relative abundances of bacteriomes varied between soil samples collected from rooftop and surface gardens in the Dhaka and Gazipur districts of Bangladesh. The study revealed a notably higher bacterial diversity in surface garden soils (DSG = 717, GSG = 750 species) compared to rooftop garden soils (DRG = 699, GRG = 730), with a high overlap (87.55%) in species (**Fig 6A**), indicating a shared core bacteriome.

### 3.3 Dominance of *Bacillus* species in the garden soils of Bangladesh

A total of 41 *Bacillus* species/strains were identified in this study, of which 58.54% were shared across DRG, DSG, GRG, and GSG soil types (S4 Fig.). Across these environments, *Bacillus* species emerged as the dominant genus, consistently comprising over 53% of the total bacteriome communities (**Fig 6B**, S1 Data). Notably, *B. paralicheniformis* (28.3%), *B. licheniformis* (25.2%) and *B. cereus sensu lato* (8.567%) were most abundant in DRG soils. DSG soils showed greater species richness and more even distribution, with dominant species including *B. cereus sensu lato* (16.0%), *B. agri* (12.1%), and *F. thermophilum* (11.4%), along with *B. smithii* (8.49%), *B. safensis* (6.01%), *B. stratosphericus* (5.10%), *B. licheniformis* (4.20%), *B. altitudinis* (3.13%), and *B. pumilus* (3.08%). In GRG soils, *B. cereus sensu lato* (42.4%), *B. agri* (11.5%), *B. altitudinis* (5.43%), and *B. subtilis* (2.81%) were predominant. GSG soils were enriched with *B. stratosphericus* (14.6%), *B. licheniformis* (12.7%), *B. safensis* (9.7%), *B. altitudinis* (8.8%), and *B. pumilus* (5.1%) (**Fig 6B**, S1 Data). Unique associations were observed in specific soil samples: GSG soils were uniquely associated with three species/strains (*Rhodanobacter lindaniclasticus*, *Paenibacillus* sp. YN15, and *Acidovorax kalamii*), while DSG soils were uniquely linked to *B. cereus* group sp. Bc222 (S1 Data). Furthermore, most species identified in each sample were represented by a single strain (**Fig 6B**, S1 Data). Although the remaining microbial species exhibited relatively low abundances (< 2.5%), their distribution varied significantly across the four metagenomes (DRG, DSG, GRG, and GSG) (**Fig 6B**, S1 Data).

### 3.4 KEGG-based metabolic profiling of garden soils bacteriomes

**Fig 7:**
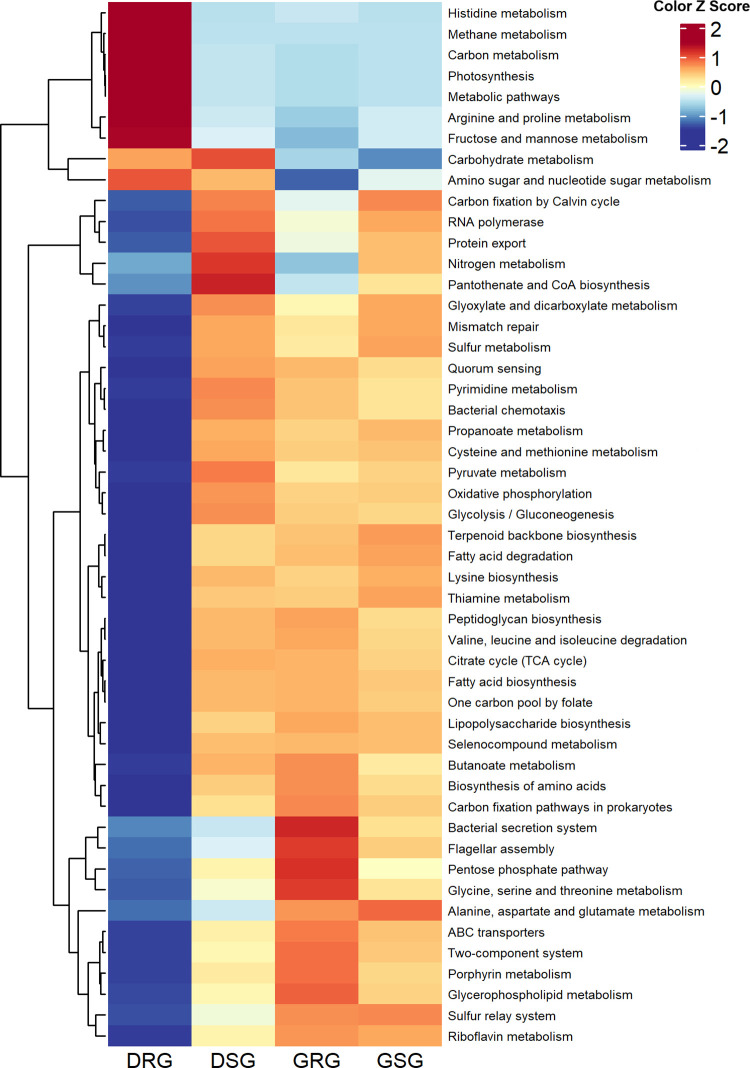
KEGG functional profile of garden soil microbiomes. The heatmap illustrates the relative abundance of the top 50 KEGG functional pathways identified in Dhaka rooftop garden (DRG), Dhaka surface garden (DSG), Gazipur rooftop garden (GRG) and Gazipur surface garden (GSG) soil microbiomes. The color bar at the top represents the relative abundance of the KEGG functional pathways (at level 3) in the corresponding sample groups. The color Z score indicate the presence and completeness of each pathway, with values ranging from −2 (lowest abundance) to 2 (highest abundance). The red color indicates the more abundant patterns, while blue cells account for less abundant pathways in that particular group. Hierarchical clustering highlights similarities and differences in metabolic potential among groups and individuals.

The KEGG functional profiling demonstrated marked metabolic stratification among garden soil bacteriomes across the study locations. The top three most abundant pathways across all groups were carbohydrate metabolism (DRG: 10.75%, DSG: 11.07%, GRG: 9.69%, GSG: 9.30%), ABC transporters (3.56–4.33%), and biosynthesis of amino acids (2.41–4.30%) (**Fig 7**, S2 Data). The most striking differences were observed in photosynthesis, which was exceptionally high in DRG (8.40%) but low in DSG, GRG and GSG garden soils (1.27–1.60%). Similarly, methane metabolism (8.62%) and carbon metabolism (7.02%) were highly enriched in DRG. In contrast, several core metabolic and regulatory pathways were more prominent in the other three sites: oxidative phosphorylation (DSG: 4.08%, GRG: 3.75%, GSG: 3.79%), two-component system (3.24–3.73%), and pyrimidine metabolism (2.97–3.25%) were all substantially higher in DSG, GRG, and GSG compared to DRG. Pathways like glycolysis/gluconeogenesis and quorum sensing also followed this pattern, with approximately two- to threefold higher relative abundances in DSG, GRG and GSG garden soils compared to DRG soils (**Fig 7**, S2 Data).

## 4. Discussion

Next-generation sequencing has transformed metagenomic research by enabling detailed analysis of microbiomes and their ecological roles in diverse ecosystems [37,53,54]. Improvements in sequencing and bioinformatics enhance taxonomic resolution and functional annotation, and facilitate the discovery of novel microbial taxa [29,31,55]. Moreover, shotgun metagenomics (WMS) reveals functional genes and metabolic pathways relevant to nutrient cycling, plant growth promotion, and the identification of enzymes and bioactive compounds with agricultural and biomedical potential [56,57]. Urban garden soils, though underexplored, are vital reservoirs of bacterial diversity with direct implications for sustainable agriculture. This study advances our understanding by characterizing how garden type (rooftop vs. surface) and geographic location (Dhaka vs. Gazipur) influence soil bacteriome composition, with a particular focus on the ecological dominance of *Bacillus*-a genus renowned for its PGP traits. Dhaka and Gazipur were selected to represent the contrast between highly urbanized and peri-urban agricultural systems in Bangladesh. Dhaka, as the most populous metropolitan area, imposes intense anthropogenic pressure and higher exposure to contaminants [58,59]. In contrast, Gazipur represents a peri-urban area with active agriculture and comparatively lower city-related stress [60,61]. Together, these sites provide a robust framework to examine microbiome variation along a critical urbanization gradient in central Bangladesh. Sampling was conducted between April and July 2024, coinciding with the main growing season when soil microbial activity and plant–soil interactions are at their peak, thereby enabling ecologically relevant community profiling [62,63].

While alpha diversity remained consistent, the beta diversity analysis revealed distinct community structuring across the groups, suggesting that location-specific selective pressures, rather than broad diversity measures, are key ecological drivers [9,64]. These overall taxonomic findings align with global studies identifying *Firmicutes* and *Proteobacteria* as dominant urban soil phyla [3,65]. The most striking finding is the high abundance of *Bacillus* species, comprising over 53% of the bacteriome across all soils-a novel observation for urban agroecosystems. This prevalence highlights the genus's ecological plasticity and adaptability. Crucially, the location-specific *Bacillus* profiles indicate distinct ecological selection pressures. For instance, the higher abundance of stress-tolerant taxa like UV-resistant *B. safensis* and radiation-resistant *B. stratosphericus* in the high-density urban core of Dhaka suggests adaptation to greater environmental stress, such as light exposure in rooftop gardens or residual urban pollutants [21,66]. The specific distribution of *Bacillus* species underscores their ecological versatility. For instance, the high abundance of *B. paralicheniformis* and *B. licheniformis* in nutrient-poor rooftop soils suggests their vital role in sustaining productivity through traits like nitrogen fixation and phosphate solubilization [14,67]. The pronounced beta-diversity clustering, coupled with uneven taxonomic distributions (including the high abundance of *B. cereus sensu lato* in GRG soils; 42.4%), appears to be driven by bioremediation and biocontrol-associated functions [68–70], are driven by variations in local management and organic inputs, including compost quality and water source, which differ between the two regions. This spatial variation in microbial communities confirms a strong environmental filter, driving the selection of specialized microbial communities. This finding is critical for highlighting the practical relevance of location-specific soil management strategies for sustainable urban agriculture. The enrichment of probiotic genera such as *Paenibacillus*, *Lysinibacillus*, and *Sphingomonas* [71–73] further supports their potential for developing microbiome-assisted, biofertilizer-based soil health strategies. The abundance of *B. licheniformis* and *B. cereus sensu lato* in nutrient-poor rooftop soils, for example, positions them as promising candidates for biofertilizer development to enhance crop resilience. *Bacillus cereus sensu lato* strains are particularly notable for their larvicidal activity through the production of toxins and secondary metabolites, providing sustained biocontrol against disease vectors [22,74–76].

The location-specific microbial patterns observed here underscore the need for tailored soil management practices. This study, while groundbreaking in its approach for this region, has several limitations. Its focus on analyzing 27 samples only from two cities and a specific time frame limits broader applicability and fails to account for seasonal variations. Furthermore, this study builds upon our prior work that utilized *16S rRNA* gene amplicon sequencing [3] on a similar set of urban garden soils and also indicated Bacillus dominance. The samples used in this study are new and were collected during a different season (Summer: April-July 2024) compared to the previous study (Monsoon: August-September 2023), with seasonal variation likely being a major driver of the observed differences in community structure. The application of shotgun metagenomics, as opposed to *16S rRNA* amplicon sequencing, provides the superior resolution necessary to confirm this dominance at the species level and to identify key taxa such as B. paralicheniformis, B. licheniformis, and B. cereus sensu lato with greater confidence, providing a more detailed understanding of the urban soil bacteriomes.

Moreover, the KEGG analysis revealed a marked functional divergence between the DRG and the other surface/rooftop soils (DSG, GRG, GSG). The DRG bacterial communities were highly specialized, showing notable enrichment in photosynthesis and methane metabolism, indicative of a unique, phototroph-driven consortium adapted to the isolated, high-light rooftop niche [68,77]. In contrast, DSG, GRG, and GSG communities exhibited a versatile, heterotroph-dominated profile, with significantly higher abundances of oxidative phosphorylation, two-component systems, and quorum sensing. This pattern reflects an adaptation to the more competitive, resource-rich surface soil environment, where efficient organic matter processing, stress response, and microbial communication are critical [78,79]. The results underscore location (rooftop vs. surface) as a primary driver of microbial metabolic strategy in urban agroecosystems.

Despite these insights, several limitations must be acknowledged. The study was restricted to two cities and a single sampling period, which may not capture seasonal or broader geographic variations. Moreover, while shotgun metagenomics provided species-level resolution, this study did not include strain isolation, non-bacterial communities (*e.g.*, archaea, viruses, bacteriophages, fungi, molds, and yeasts) or microbial network analyses. As a result, interpretations of PGP potential remain putative. Future investigations should isolate dominant strains, validate their PGP traits, and evaluate their field performance to strengthen the link between microbial ecology and sustainable urban farming [55,56]. Such efforts would bridge the gap between microbial ecology and sustainable urban farming, helping to address soil degradation in rapidly urbanizing regions.

## 5. Conclusions

This study provides a comprehensive characterization of the bacteriome in rooftop and surface garden soils of Dhaka and Gazipur districts of Bangladesh. While alpha diversity analysis indicated a similar level of bacterial richness and evenness across all samples, beta diversity analysis using PCoA and NMDS plots revealed significant differences in bacterial community composition. These distinctions were primarily driven by a combination of geographic location and garden type, suggesting that local environmental factors and management practices are key determinants of microbial community structure. At the phylum level, *Firmicutes* and *Proteobacteria* were the most dominant taxa across all samples, demonstrating their adaptability to urban and peri-urban soil environments. At the genus level, *Bacillus* emerged as the most dominant and ubiquitous taxon, consistently comprising over 53% of the total bacteriome. However, the specific species and their relative abundances within the *Bacillus* genus varied significantly between rooftop and surface gardens, highlighting a core bacteriome with a flexible, location-specific species composition. In addition, KEGG functional annotations reflected marked functional divergence, with DRG bacterial communities being specialized in phototrophic processes, whereas the other garden soils are more strongly associated with pathways related to energy metabolism, cellular communication, and biosynthetic activity. The prevalence of resilient, spore-forming bacteria like *Bacillus* in these urban soil environments underscores their ecological importance and potential for biotechnological applications.

## Supporting information

S1 Data Taxonomic information of the soil microbiomes (phyla to species/strain levels) identified in in Dhaka rooftop garden (DRG), Dhaka surface garden (DSG), Gazipur rooftop garden (GRG) and Gazipur surface garden (GSG). (XLSX)

S2 Data KEGG functional pathways identified in Dhaka rooftop garden (DRG), Dhaka surface garden (DSG), Gazipur rooftop garden (GRG) and Gazipur surface garden (GSG) soil bacteriomes. (XLSX)

S1 Fig Alpha diversity of soil bacterial communities in rooftop and surface gardens across Dhaka and Gazipur, Bangladesh. The indices include Chao1 and Observed (measuring richness), and Shannon and Simpson (measuring richness and evenness). Values are presented for analyses conducted at both the (A) Phylum and (B) Order taxonomic levels. Boxplots display the results, and pairwise Kruskal-Wallis tests assess statistical differences, with p-values indicating no significance differences (*p* > 0.05).
(DOCX)

S2 Fig Beta diversity of soil bacterial communities in rooftop and surface gardens across Dhaka and Gazipur, Bangladesh. Values are presented for analyses conducted at both the (A) Phylum and (B) Order taxonomic levels. The data is visualized through principal coordinate analysis (PCoA) measured using Bray-Curtis dissimilarity, where samples are color-coded by garden location, with ellipses representing group clustering. The percentage of variation along the X and Y axes highlights the extent to which garden location shape microbial diversity.
(DOCX)

S3 Fig Phylum level taxonomic profile of microbiomes. Bar plots showing the average relative abundances of the bacteria at phylum level in the rooftop garden soil and surface garden soil samples in both Dhaka and Gazipur districts of Bangladesh. The distribution and relative abundance of the bacterial phyla in the study metagenomes are also available in Data S1.
(DOCX)

S4 Fig Venn diagrams showing the unique and shared composition of *Bacillus* species in Dhaka rooftop garden (DRG), Dhaka surface garden (DSG), Gazipur rooftop garden (GRG) and Gazipur surface garden (GSG) soil samples. Shared species taxa are highlighted in red circle.
(DOCX)

S1 Table Study sample information, SRA accession numbers of the shotgun whole metagenome sequences and taxonomic units (N = 766) mapped against microbial taxa. (DOCX)
