# Supplementary material for: Shotgun metagenomic profiling reveals Bacillus-dominated bacterial communities in urban rooftop and surface garden soils of Bangladesh
Source: PLoS One. 2026 Mar 6;21(3):e0344114. doi: 10.1371/journal.pone.0344114 (PMC12965560; doi:10.1371/journal.pone.0344114)
Supplement: S1 Fig — The indices include Chao1 and Observed (measuring richness), and Shannon and Simpson (measuring richness and evenness). Values are presented for analyses conducted at both the (A) Phylum and (B) Order taxonomic levels. Boxplots display the results, and pairwise Kruskal-Wallis tests assess statistical differences, with p-values indicating no significance differences (p > 0.05). (DOCX) [file pone.0344114.s003.docx]

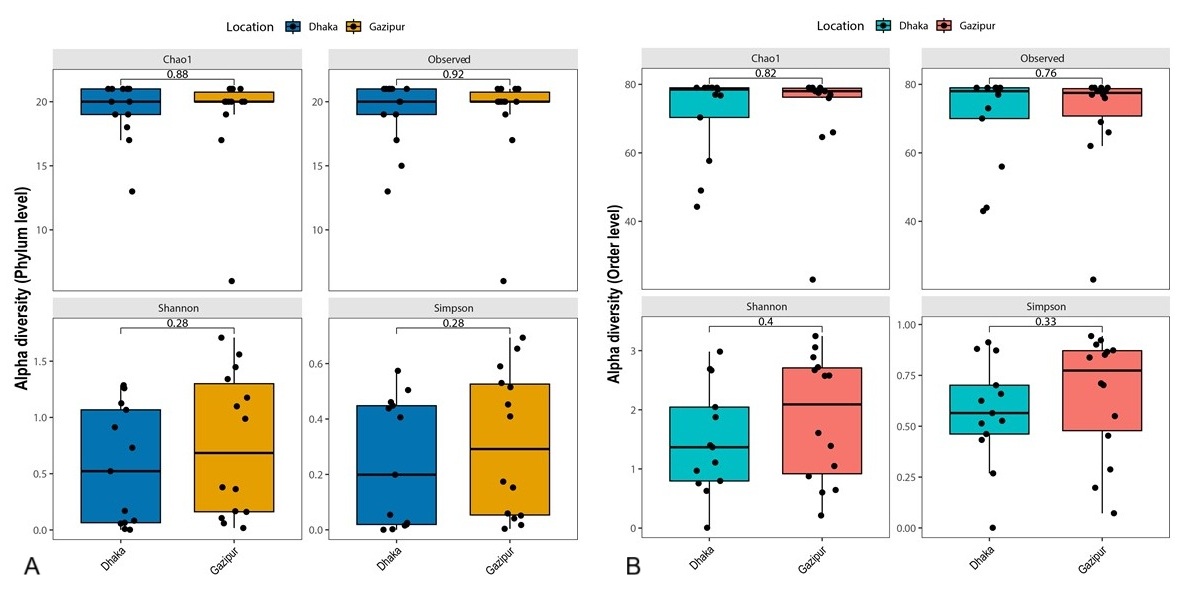


**S1 Fig.** Alpha diversity of soil bacterial communities in rooftop and surface gardens across Dhaka and Gazipur, Bangladesh. The indices include Chao1 and Observed (measuring richness), and Shannon and Simpson (measuring richness and evenness). Values are presented for analyses conducted at both the (A) Phylum and (B) Order taxonomic levels. Boxplots display the results, and pairwise Kruskal-Wallis tests assess statistical differences, with p-values indicating no significance differences (*p* > 0.05).
