# Supplementary material for: Shotgun metagenomic profiling reveals Bacillus-dominated bacterial communities in urban rooftop and surface garden soils of Bangladesh
Source: PLoS One. 2026 Mar 6;21(3):e0344114. doi: 10.1371/journal.pone.0344114 (PMC12965560; doi:10.1371/journal.pone.0344114)
Supplement: S2 Fig — Values are presented for analyses conducted at both the (A) Phylum and (B) Order taxonomic levels. The data is visualized through principal coordinate analysis (PCoA) measured using Bray-Curtis dissimilarity, where samples are color-coded by garden location, with ellipses representing group clustering. The percentage of variation along the X and Y axes highlights the extent to which garden location shape microbial diversity. (DOCX) [file pone.0344114.s004.docx]

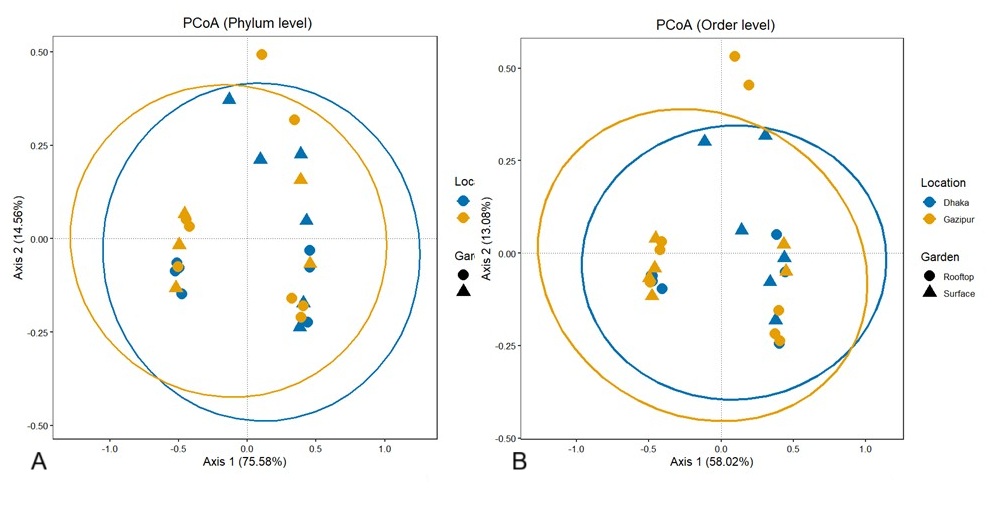


**S2 Fig.** Beta diversity of soil bacterial communities in rooftop and surface gardens across Dhaka and Gazipur, Bangladesh. Values are presented for analyses conducted at both the (A) Phylum and (B) Order taxonomic levels. The data is visualized through principal coordinate analysis (PCoA) measured using Bray-Curtis dissimilarity, where samples are color-coded by garden location, with ellipses representing group clustering. The percentage of variation along the X and Y axes highlights the extent to which garden location shape microbial diversity.
