# Supplementary material for: Shotgun metagenomic profiling reveals Bacillus-dominated bacterial communities in urban rooftop and surface garden soils of Bangladesh
Source: PLoS One. 2026 Mar 6;21(3):e0344114. doi: 10.1371/journal.pone.0344114 (PMC12965560; doi:10.1371/journal.pone.0344114)
Supplement: S3 Fig — Bar plots showing the average relative abundances of the bacteria at phylum level in the rooftop garden soil and surface garden soil samples in both Dhaka and Gazipur districts of Bangladesh. The distribution and relative abundance of the bacterial phyla in the study metagenomes are also available in Data S1. (DOCX) [file pone.0344114.s005.docx]

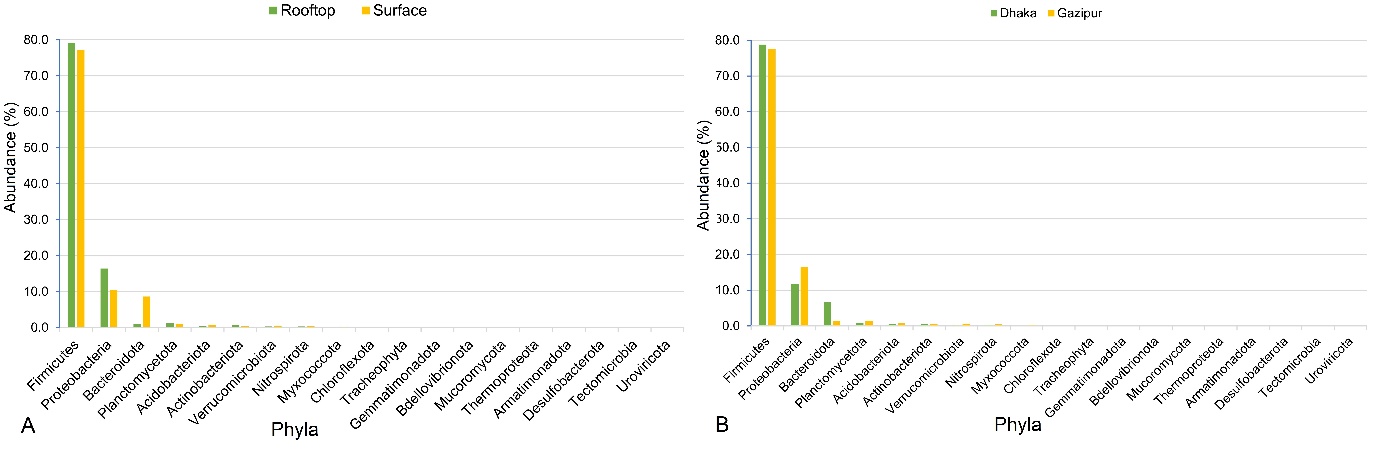


**S3 Fig.** Phylum level taxonomic profile of microbiomes. Bar plots showing the average relative abundances of the bacteria at phylum level in the rooftop garden soil and surface garden soil samples in both Dhaka and Gazipur districts of Bangladesh. The distribution and relative abundance of the bacterial phyla in the study metagenomes are also available in Data S1.
