# Supplementary material for: Shotgun metagenomic profiling reveals Bacillus-dominated bacterial communities in urban rooftop and surface garden soils of Bangladesh
Source: PLoS One. 2026 Mar 6;21(3):e0344114. doi: 10.1371/journal.pone.0344114 (PMC12965560; doi:10.1371/journal.pone.0344114)
Supplement: S4 Fig — Shared species taxa are highlighted in red circle. (DOCX) [file pone.0344114.s006.docx]

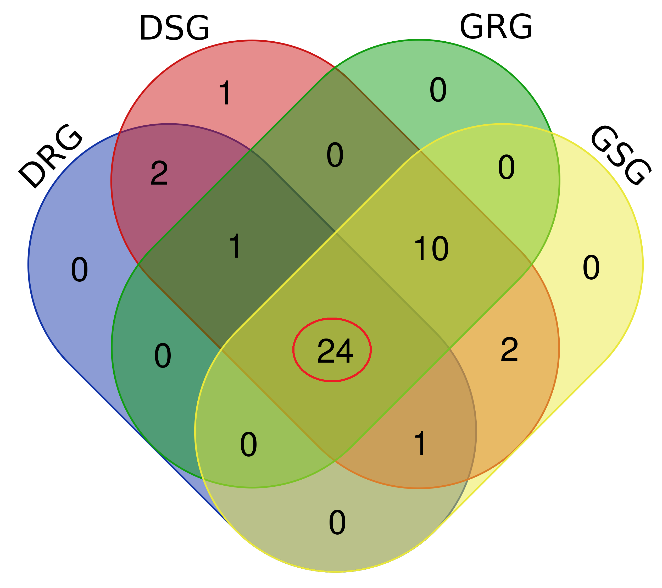


**S4 Fig.** Venn diagrams showing the unique and shared composition of *Bacillus* species in Dhaka rooftop garden (DRG), Dhaka surface garden (DSG), Gazipur rooftop garden (GRG) and Gazipur surface garden (GSG) soil samples. Shared species taxa are highlighted in red circle.
