# Supplementary material for: Shotgun metagenomic profiling reveals Bacillus-dominated bacterial communities in urban rooftop and surface garden soils of Bangladesh
Source: PLoS One. 2026 Mar 6;21(3):e0344114. doi: 10.1371/journal.pone.0344114 (PMC12965560; doi:10.1371/journal.pone.0344114)
Supplement: S1 Table — (DOCX) [file pone.0344114.s007.docx]

**S1 Table.** Study sample information, SRA accession numbers of the shotgun whole metagenome sequences and taxonomic units (N = 766) mapped against microbial taxa.

| **Sl. No.** | **Sample ID** | **Collection site** | **Coordinates** | **Source** | **No. of raw reads (before trimming)** | **No. of quality reads (after trimming)** | **No. of mapped reads** | **GC Content (%) of mapped reads** | **No. of**  **taxonomic unit** | **SRA accessions** |
| --- | --- | --- | --- | --- | --- | --- | --- | --- | --- | --- |
| 1 | DR1 | DNCC, Bangladesh | 23.78° N, 90.41° E | DRG | 59967810 | 59559296 | 1393674 | 39.46 | 180 | SRR32835637 |
| 2 | DR2 | DNCC, Bangladesh | 23.78° N, 90.41° E | DRG | 60662638 | 59298504 | 390962 | 62.04 | 756 | SRR32835631 |
| 3 | DR3 | DNCC, Bangladesh | 23.78° N, 90.41° E | DRG | 48820994 | 46575074 | 452424 | 61.78 | 748 | SRR32835620 |
| 4 | DR4 | DNCC, Bangladesh | 23.78° N, 90.41° E | DRG | 44284430 | 43462174 | 513703 | 62.94 | 753 | SRR32835612 |
| 5 | DR6 | DNCC, Bangladesh | 23.78° N, 90.41° E | DRG | 47117638 | 46222048 | 611602 | 63.54 | 749 | SRR32835611 |
| 6 | DR7 | DNCC, Bangladesh | 23.78° N, 90.41° E | DRG | 49250464 | 48858846 | 1546138 | 44.44 | 344 | SRR32835636 |
| 7 | DR9 | DNCC, Bangladesh | 23.78° N, 90.41° E | DRG | 44994610 | 44651424 | 2446518 | 46.56 | 207 | SRR32835635 |
| 8 | DS1 | DNCC, Bangladesh | 23.78° N, 90.41° E | DSG | 51863182 | 51380808 | 2738856 | 42.56 | 715 | SRR32835634 |
| 9 | DS2 | DNCC, Bangladesh | 23.78° N, 90.41° E | DSG | 43858724 | 43042600 | 839739 | 55.96 | 752 | SRR32835633 |
| 10 | DS3 | DNCC, Bangladesh | 23.78° N, 90.41° E | DSG | 47000772 | 46186496 | 404485 | 61.5 | 746 | SRR32835632 |
| 11 | DS4 | DNCC, Bangladesh | 23.78° N, 90.41° E | DSG | 56191782 | 55699876 | 2049273 | 51.18 | 730 | SRR32835630 |
| 12 | DS5 | DNCC, Bangladesh | 23.78° N, 90.41° E | DSG | 61243454 | 60757998 | 800749 | 42.89 | 505 | SRR32835629 |
| 13 | DS6 | DNCC, Bangladesh | 23.78° N, 90.41° E | DSG | 42711488 | 42475102 | 1176498 | 40.31 | 547 | SRR32835628 |
| 14 | GR1 | GCC, Bangladesh | 23.99° N, 90.38° E | GRG | 44554198 | 43572430 | 586690 | 60.78 | 756 | SRR32835626 |
| 15 | GR2 | GCC, Bangladesh | 23.99° N, 90.38° E | GRG | 50425924 | 49278262 | 488199 | 62.43 | 751 | SRR32835627 |
| 16 | GR3 | GCC, Bangladesh | 23.99° N, 90.38° E | GRG | 50775720 | 50397022 | 2099824 | 45.54 | 691 | SRR32835625 |
| 17 | GR4 | GCC, Bangladesh | 23.99° N, 90.38° E | GRG | 42067910 | 41566356 | 285853 | 38.89 | 432 | SRR32835624 |
| 18 | GR5 | GCC, Bangladesh | 23.99° N, 90.38° E | GRG | 43335504 | 42779038 | 1919058 | 41.78 | 712 | SRR32835623 |
| 19 | GR6 | GCC, Bangladesh | 23.99° N, 90.38° E | GRG | 43906704 | 43422746 | 640775 | 41.71 | 405 | SRR32835622 |
| 20 | GR7 | GCC, Bangladesh | 23.99° N, 90.38° E | GRG | 45894154 | 45222888 | 208619 | 64.29 | 733 | SRR32835621 |
| 21 | GR9 | GCC, Bangladesh | 23.99° N, 90.38° E | GRG | 44698694 | 44209214 | 2347772 | 44.23 | 687 | SRR32835619 |
| 22 | GS1 | GCC, Bangladesh | 23.99° N, 90.38° E | GSG | 66333510 | 65834502 | 936620 | 39.03 | 84 | SRR32835618 |
| 23 | GS2 | GCC, Bangladesh | 23.99° N, 90.38° E | GSG | 60495574 | 59960662 | 1510564 | 42.49 | 409 | SRR32835617 |
| 24 | GS3 | GCC, Bangladesh | 23.99° N, 90.38° E | GSG | 50210436 | 49409548 | 348061 | 60.37 | 756 | SRR32835615 |
| 25 | GS4 | GCC, Bangladesh | 23.99° N, 90.38° E | GSG | 46783876 | 45616480 | 240224 | 63.87 | 723 | SRR32835616 |
| 26 | GS5 | GCC, Bangladesh | 23.99° N, 90.38° E | GSG | 47019134 | 46116762 | 500388 | 59.88 | 746 | SRR32835614 |
| 27 | GS7 | GCC, Bangladesh | 23.99° N, 90.38° E | GSG | 50644536 | 49551752 | 332346 | 61.08 | 755 | SRR32835613 |
| Total | | | | | 1345113860 | 1325107908 | 27809614 |  | |  |
| Average | | | | | 49819031.85 | 49078070.67 | 1029985.704 | 51.91 | 606.37 |  |

DNCC: Dhaka North City Corporation, GCC: Gazipur City Corporation, DRG: Dhaka Rooftop Garden, DSG: Dhaka Surface Garden, GRG: Gazipur Rooftop Garden,

GSG: Gazipur Surface Garden, DR: Dhaka Rooftop, DS: Dhaka Surface, GR: Gazipur Rooftop, and GS: Gazipur Surface.
